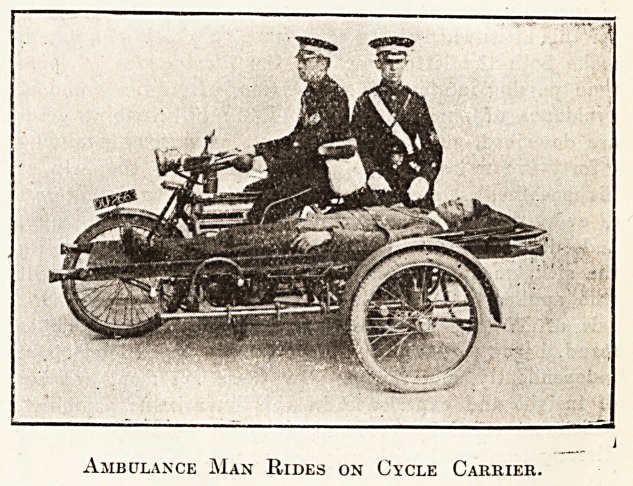# The Motor-Cycle Ambulance

**Published:** 1914-10-10

**Authors:** 


### The Motor-Cycle Ambulance.

A little-known type of motor ambulance is the motor-cycle and stretcher side-car, which has recently made its appearance, and our illustrations give a glance at two such outfits which have recently been seen.

The "Gloria" ambulance side-stretcher outfit con-

"Gloria" Ambulance. Side Stretcher Combination."

sists of a specially designed chassis, with a 26-inch by $2\frac{1}{4}$-inch wheel fitted with a 26-inch by $2\frac{3}{8}$-inch tyre, which carries the stretcher, patent spring suspension being employed, which, in addition to the usual cee springs, is used to insulate the patient from any road shock. Specially designed cups are attached to the springs of the chassis, into which cones fixed to the sides of the stretcher are made to fit, a device which enables the stretcher to be attached or detached with ease in a few seconds. The cost of the outfit with stretcher is £25. Another interesting outfit is the "F.N.," manufactured by "F.N." Ltd. It consists of a 7-h.p. 3-speed

Ambulance Man Rides on Cycle Carrier.

4-cylinder motor-cycle, to which is attached a side-car chassis, coupled with four couplings; the whole of the front of the frame of the machine from top head lug down to the cradle lug being in one piece, and drop forged. The wheel is 26 inches by $2\frac{1}{2}$ inches, fitted with 26-inch by $2\frac{1}{2}$-inch beaded tyre, and the suspension is by two-leaf coil springs at rear $1\frac{1}{4}$ inch wide, and front springs of single leaf.

The stretcher is attached by four bolts. The cost of the outfit, apart from motor-cycle but including stretcher, which is provided with straps and hood, is £15.

## Figures and Tables

**Figure f1:**
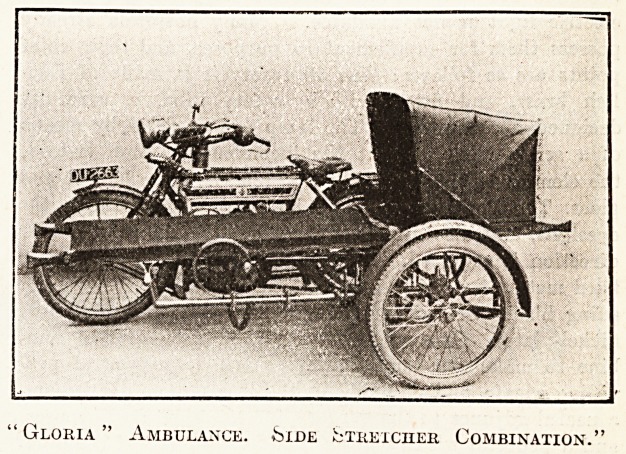


**Figure f2:**